# Seroprevalence of IgG and IgM Antibodies against *Toxoplasma gondii* in Dogs in Greece

**DOI:** 10.3390/vetsci11080339

**Published:** 2024-07-27

**Authors:** Georgios Sioutas, Athanasios I. Gelasakis, Isaia Symeonidou, Constantina N. Tsokana, Panagiotis Alevizos, Dimitra Bitchava, Elias Papadopoulos

**Affiliations:** 1 Laboratory of Parasitology and Parasitic Diseases, School of Veterinary Medicine, Faculty of Health Sciences, Aristotle University of Thessaloniki, 54124 Thessaloniki, Greece; gsioutas@vet.auth.gr (G.S.); isaia@vet.auth.gr (I.S.); nt.tsokana@live.com (C.N.T.); 2 Laboratory of Anatomy and Physiology of Farm Animals, Department of Animal Science, School of Animal Biosciences, Agricultural University of Athens, 11855 Athens, Greece; gelasakis@aua.gr; 3 Vet in Progress Plus, Veterinary Laboratories, Agia Paraskevi, 15343 Attiki, Greece; p.alevizos@vetinprogress.gr (P.A.); information@vetinprogress.gr (D.B.)

**Keywords:** dogs, Greece, IFAT, IgG, IgM, public health, rural environment, seroprevalence, *Toxoplasma gondii*, toxoplasmosis

## Abstract

**Simple Summary:**

Toxoplasmosis is a disease caused by the parasite *Toxoplasma gondii,* which can infect humans and animals, including dogs. Dogs become infected by accidentally ingesting the parasite’s oocysts from the environment and eating infected rodents or other small animals. Following infection, dogs develop antibodies against *T. gondii.* The current study investigated the percentage of dogs living in Greece that have antibodies against the parasite as an indirect way to assess the level of environmental contamination with *T. gondii* oocysts. Blood samples were collected from 1282 dogs living in both urban and rural regions of Greece, including Attica and Thessaloniki. Overall, 47.6% of the dogs had antibodies against the parasite, indicating they had been exposed to *T. gondii*. Seropositive dogs were more commonly found in rural areas (53.8%) than in urban areas (43.9%) (*p* < 0.001). The findings of this study suggest that *T. gondii* infection is widespread in dogs across Greece, especially in rural regions, and the parasite is highly present in the environment, posing a potential risk for human exposure in these areas. Control measures are necessary to prevent dogs from being infected and to reduce this risk for dog owners. Dog owners should also follow basic hygiene practices, like washing their hands after petting their dogs, to protect themselves.

**Abstract:**

Toxoplasmosis, caused by the protozoan *Toxoplasma gondii*, is a zoonotic disease that affects various animal species, including dogs, that can serve as sentinels for indirectly estimating the environmental contamination. The current study aimed to determine the seroprevalence of *T. gondii* IgG and IgM antibodies in dogs across different regions of Greece and assess their living area as a potential risk factor. In total, 1282 blood samples were collected from dogs in urban and rural areas of Greece, including Attica and Thessaloniki. Serum samples were tested for *T. gondii*-specific IgG and IgM antibodies using an indirect immunofluorescent antibody test. A chi-square test was performed to assess the association between seropositivity for *T. gondii* and geographical location (urban/rural). The overall *T. gondii* seroprevalence was 47.6%, while the seroprevalence of IgG and IgM antibodies was 34.3% and 22.2%, respectively. Dogs from rural areas exhibited a significantly higher seroprevalence (53.8%) than those from urban areas (43.9%) (*p* < 0.001), with the estimated odds ratio being equal to 1.49 (95% CI, 1.18 to 1.65) and the relative risk increased by 22.4%. Dogs in Greece are highly exposed to *T. gondii*, particularly in rural areas. Measures to prevent canine infections are necessary, and basic hygiene practices, such as hand washing after petting dogs, are required to reduce human infection risk and safeguard public health.

## 1. Introduction

*Toxoplasma gondii* is a protozoon that causes the zoonotic disease toxoplasmosis, affecting virtually all warm-blooded animals, including humans, cats, and dogs [1]. Felines are the sole definitive hosts, shedding oocysts with their faeces, while many other animal species serve as intermediate hosts [1]. Transmission occurs mainly via the ingestion of sporulated oocysts from the environment or bradyzoites in tissue cysts of raw or undercooked meat, and vertically from the mother to the foetus [2,3,4,5].

Canine toxoplasmosis is rarely a primary disease. Following infection, dogs seroconvert, while most remain asymptomatic and do not develop pathological lesions [6,7,8]. Clinical toxoplasmosis is mainly associated with immunosuppression and co-infection with other canine pathogens such as *Ehrlichia canis* or the canine distemper virus (CDV) [8,9,10]. Similarly, young dogs, immunosuppressed dogs, or those undergoing corticosteroid treatment or chemotherapy are more prone to develop clinical disease [8,9,10].

When dogs exhibit symptoms, they are generalised or typically concern the lungs, central nervous system (CNS), muscles, or alimentary tract [9]. Infection of the CNS can persist for many days up to months, while infection of the pulmonary system or liver can prove fatal in just seven days [9]. Generalised clinical signs are more common in dogs under 12 months of age, including pyrexia, dyspnoea, diarrhoea, tonsil inflammation, hepatocellular icterus, and vomitus [9]. Cardiac infections are usually asymptomatic, but older dogs can show congestive heart failure and cardiac arrhythmias [9]. As for infections during pregnancy, they can lead bitches to abort [3,5,11].

Toxoplasmosis is more prevalent in cats than dogs, with the latter more frequently affected by neosporosis. In fact, until the discovery of *Neospora caninum* in 1988, many cases of neosporosis in dogs were misdiagnosed as canine toxoplasmosis [9,12]. Both protozooses have similar clinical pictures and low morbidity and fatality rates [8,9]. Furthermore, clinical toxoplasmosis cases in dogs may have decreased in the last decades due to the routine vaccination of dogs for CDV [9].

Diagnosis of toxoplasmosis can be achieved with different methods, including the detection of specific anti-*T. gondii* IgG and IgM antibodies in the dog’s serum [13]. IgG antibodies indicate a chronic infection and persist throughout the dog’s life, while IgM indicate a recent infection [9]. Detecting both IgG and IgM in dogs provides a clearer picture regarding recent and past *T. gondii* infections [14]. The indirect immunofluorescent antibody test (IFAT) is one of the most commonly used methods for detecting IgG antibodies in dogs and other animals and is also used to detect IgM antibodies [1,13,15]. Until now, most seroprevalence studies for *T. gondii* have employed IFAT due to its high diagnostic accuracy [1,10]. In fact, IFAT is highly specific, and if appropriate cut-off thresholds are utilised, there is no cross-reaction with antibodies against other protozoans, such as *N. caninum* [1,10]. It is worth noting that no serological test can provide a conclusive diagnosis of toxoplasmosis, and there is no association between antibody titers and the intensity of the symptoms [9].

Infection in dogs is of epidemiological relevance. Estimating the levels of environmental contamination with *T. gondii* oocysts can be directly performed by measuring the number of oocysts in the environment. However, this approach remains challenging and impractical [16,17]. On the other hand, since dogs that have been exposed to *T. gondii* seroconvert, assessing the seroprevalence in this animal species can be used as an indirect indication of *T. gondii* presence in a specific area. Seropositivity in dogs can also be used to evaluate human infection risk in an area since it is suggestive of the dogs’ previous exposure to *T. gondii* in their living environment. This is relevant to human infection risk because dogs and humans usually live in close proximity. Moreover, studies have shown that seroprevalence rates in human and canine populations are positively associated [17,18,19,20,21].

Furthermore, investigating the *T. gondii* seroprevalence in dogs is essential since they are the most common pets in Greek households [22]. Oocyst production does not occur in dogs because they are dead-end intermediate hosts for this protozoon [23]. However, they can mechanically carry and spread *T. gondii* oocysts through two routes [6,24,25]. Firstly, they like rolling in soil, foul-smelling substances, and cat faeces (xenosmophilia), which may contain oocysts. This way, dogs pick oocysts up on their fur and carry them inside the house. As a result, humans who pet them may be exposed to *T. gondii* oocysts [6,24,25]. Secondly, dogs may contribute to environmental contamination by excreting oocysts following ingestion of infected cat faeces during coprophagy, a typical canine behaviour [6,20,24,25,26]. However, the epidemiological significance of the mechanical transmission of *T. gondii* by dogs may be questioned since it has been shown that oocysts do not sporulate on the dog’s coat, probably due to unfavourable temperature and humidity conditions [6]. On the other hand, viable oocysts have been recovered from dog faeces under experimental [6] and natural [26] infection conditions. These findings justify the recommendation of hand washing after [27] contact with dogs or dog faeces to prevent *T. gondii* infections in humans.

In addition to being the most popular pet animal in Greece [22], a significant part of the canine population is livestock guarding dogs and hunting dogs in rural and mountainous regions. Concurrently, there are many stray dogs in both rural and urban areas. Given the large and diverse canine population in Greece, the epidemiological role of dogs in a poorly studied area, as well as the lack of previous studies investigating the prevalence of antibodies against *T. gondii* in dogs in Greece [28], the current study aimed to (a) estimate the seroprevalence of IgG and IgM anti-*T. gondii* antibodies in dogs in the country, and (b) investigate the role of dogs’ living environment (urban vs. rural areas) as a potential risk factor for their exposure to *T. gondii*.

## 2. Materials and Methods

### 2.1. Blood Sampling and Investigation Area

In 2023, 1282 blood samples were collected from dogs (*Canis lupus familiaris*) living in Attica, Thessaloniki, and other areas of Greece. Attica and Thessaloniki represent Greece’s two most densely populated urban areas, with approximately 4.9 million people (47% of the Greek population) [29], while all the other sampling regions were rural areas.

All dogs were selected using simple random sampling, meaning that a subset of dogs was randomly selected from the total number of dogs admitted to the various private veterinary practices for routine examinations involving blood sampling. They were apparently healthy, older than 6 months old, and belonged to different breeds and both sexes. The cephalic, jugular, or saphenous vein was punctured in each dog to draw 2 mL of blood, which was subsequently placed into tubes with a clot activator. After allowing the blood to clot, the tubes were centrifuged at 1500× *g* for 10 min to obtain the serum. Serum samples were stored at −20 °C until further examination.

### 2.2. Serological Antibody Testing

Each sample was examined using a commercially available indirect immunofluorescent antibody test kit (IFAT) to detect IgG and IgM antibodies. Sera were diluted 2-fold from 1:100 to 1:1600 in Phosphate Buffer Solution (PBS) (P3812-10PAK, Sigma-Aldrich, St. Louis, MO, USA) and tested on commercial coated slides with *T. gondii* antigens (MegaFLUO^®^ TOXOPLASMA g., Art.-No. 826S50FK1, MEGACOR, Hörbranz, Austria) using manufacturer’s instructions, except replacing conjugate with anti-dog IgG FITC conjugated antibody (F7884-2ml, Sigma-Aldrich, St. Louis, MO, USA) diluted 1:200 in PBS. The anti-canine IgM FITC conjugated antibody (CJ-F-CANM-AP-10ML, VMRD, Pullman, WA, USA) was used for the IgM detection. Tests were evaluated under a fluorescence microscope with a filter system for FITC (iScope, Euromex, Arnhem, The Netherlands). The cut-offs employed were 1/200 for IgG and 1/100 for IgM. Each IFAT was carried out by the same experienced person who was blinded to each sample’s origin. Both positive and negative controls included in the kit were used in each analysis.

### 2.3. Statistical Analyses

For the estimation of the prevalence rates and their 95% confidence intervals (CI 95%), the Epitools (ausvet.com.au) and the Wilson score interval methods were used. Chi-square tests were performed using SPSS v23, with Cramér’s V values (φc), odds ratios, and relative risks being estimated to assess the association between the region (urban/rural) and the serological status with regard to IgG, IgM, or either of them. Statistical significance was set at the 0.05 level.

## 3. Results

The overall seropositivity in either IgG and/or IgM was 47.6% (610/1282, 95% CI: 44.9 to 50.3%), with 34.3% (440/1282, 95% CI: 31.8 to 37.0%) of samples being seropositive for IgG and 22.2% (284/1282, 95% CI: 20.0 to 24.5%) of samples seropositive for IgM. In addition, 8.9% (114/1282, 95% CI: 7.5 to 10.6%) of dogs were seropositive for both IgG and IgM antibodies. The IgG titers ranged from 1/100 to 1/1600, and the IgM titers ranged from 1/100 to 1/800.

**Figure 1:**
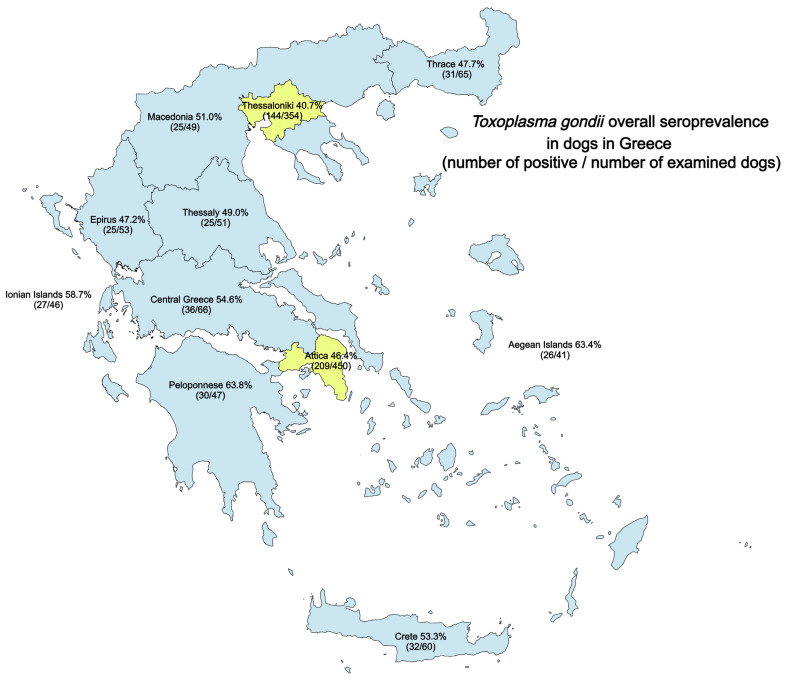
*Toxoplasma gondii* overall seroprevalence (IgG and/or IgM) in dogs from different geographic regions of Greece. The parentheses include the numbers of positive/numbers of examined dogs in each region. Thessaloniki and Attica represent the urban areas of the current study and are painted in a different colour.

Regarding sampling areas, the seropositivity in Attica was 46.4% (209/450, 95% CI: 41.9 to 51.1%) in Thessaloniki, 40.7% (144/354, 95% CI: 35.7 to 45.9%), and 53.8% (257/478, 95% CI: 49.3 to 58.2%) in the rest of rural Greece when considering either IgG or IgM. The seroprevalence for each antibody test in each area is summarised in Table 1, and the overall seroprevalence rates (IgG and/or IgM) in the different regions examined are illustrated in Figure 1. When considering urban and rural areas, dogs originating from urban areas had a seroprevalence of 43.9% (353/804, 95% CI: 40.5 to 47.4%), while in rural areas, seropositivity was 53.8% (257/478, 95% CI: 49.3 to 58.2%) in either IgM or IgG tests. In rural areas, dogs were more likely to be found seropositive for antibodies against *T. gondii* (IgG, IgM, or both) (ϕc = 0.091, and *p* < 0.001) compared to dogs in urban areas, with the estimated odds ratio being equal to 1.49 (95% CI, 1.18 to 1.65) and the relative risk increased by 22.4%. When seropositivity was separately considered for IgM and IgG, no significant differences were observed concerning IgM positivity between dogs in urban and rural areas, contrary to IgG positivity, where in rural areas, dogs were more likely to be found positive (ϕc = 0.064, and *p* = 0.021) compared to dogs in urban areas with the estimated odds ratio being equal to 1.32 (95% CI, 1.04 to 1.67) and the relative risk increased by 19.8%.

## 4. Discussion

In the current study, the overall *T. gondii* seropositivity of dogs was 47.6%, indicating that a large percentage of dogs in Greece have been exposed to *T. gondii*. Canine seroprevalence rates in similar studies from other countries range from 1.0% in China [10] to 98.0% in Egypt [30]. In comparison to nearby countries, dogs in Albania and Turkey displayed slightly higher overall seropositivity rates than in the current study, at 51.7% (owned dogs) and 54.3% (unspecified ownership), respectively [31]. The canine seroprevalence in some other Mediterranean countries has been estimated at 29.2% in a recent survey in Italy (owned dogs) [32] and at 58.7% in a study in Spain (kennel dogs) [33]. Seropositivity in dogs from different studies can vary because of different diagnostic methods, serum dilutions employed, sampling area climate, dog origin (stray, client-owned, or hunting dogs), the study inclusion criteria, and the oocyst environmental contamination levels [10,14,34,35].

Anti-*T. gondii* IgG antibodies were detected in 34.3% of dogs, while 22.2% were seropositive for IgM. The lower seroprevalence of IgM compared to IgG antibodies is unsurprising because, as mentioned above, IgM immunoglobulins in dogs persist for a couple of months post-infection [36], indicating an acute/recent or active infection [9]. In contrast, IgG antibodies can be detected lifelong after exposure, indicating a chronic infection [9]. Therefore, more dogs are expected to be seropositive for IgG than IgM antibodies. Our findings are in concordance with previous seroprevalence studies in dogs that also demonstrated higher seropositivity for IgG than IgM anti-*T. gondii* antibodies [14,37,38,39]. The fact that 8.9% of dogs were seropositive for both IgG and IgM indicates that these dogs were recently infected with *T. gondii* a few weeks before sampling and prior to becoming seronegative for IgM. Alternatively, these dogs may have already been seropositive for IgG from an old exposure to the parasite and were recently reinfected with *T. gondii,* causing a new increase in IgM antibodies [1].

Concerning living areas, it has been established that dogs from rural regions are expected to have higher seropositivity rates than those from urban regions [1,10,14,21,35,38,40]. Generally, rural areas tend to have higher levels of environmental contamination with oocysts compared to urban ones [14,21]. This may be attributed to the fact that wild and domestic cats are more prevalent in rural regions, there is more soil where oocysts are commonly found, and also prey animals, which represent an additional infection source, are more abundant in rural areas. These conditions increase the probability of rural dogs being exposed to *T. gondii* oocysts [14]. Moreover, people living in rural regions are more likely to feed offal to their dogs, which may contain *T. gondii* tissue cysts [35]. Our findings align with the aforementioned studies, since dogs from rural areas had a significantly higher overall seroprevalence than dogs from urban areas, and they were 1.49 times more likely to be positive for *T. gondii*. Correspondingly, in an analogous study conducted on the feline population in Greece, cats in rural areas had a significantly higher infection risk than cats in urban areas [2]. Again, these similarities imply higher levels of environmental contamination with *T. gondii* oocysts in the country’s rural areas.

According to a recent report, the total number of dogs in Greece is 1.42 million, with more than half (54%, 760,500) of the dog population being stray or shelter dogs [22]. The number of dogs examined in the current study (n = 1282) is among the highest compared to similar seroprevalence surveys in dogs from other countries [10]. Therefore, extrapolating the seropositivity rate of the current study (47.6%) in the canine population in the country, it is likely that more than 650,000 dogs in Greece may have come in contact with *T. gondii* at some point during their lifetime.

*Toxoplasma gondii* seroprevalence has been investigated in other animal species in Greece, including pigs, wild boars, sheep and goats, cattle, birds, hares and most recently, cats [2,28]. The seroprevalence in dogs in the current study was higher than the previously recorded seroprevalences in all other animal species except sheep and goats [28]. Indeed, *T. gondii* seropositivity was up to 90% in sheep from specific regions and 61.3% in goats in Greece [28]. Therefore, it becomes evident that various animal species are exposed to *T. gondii* in the country, with dogs and small ruminants presenting the highest seropositivity rates. This is of specific importance for sheep and goats since infection with *T. gondii* may lead to abortions and production losses [41].

As for the seropositivity in dogs, it is a great indicator for *T. gondii* occurrence and distribution in an area. The high seropositivity reported herein indirectly reflects the environmental contamination levels of *T. gondii* oocysts in Greece [18]. In the present study, dogs living in Thessaloniki had a lower seroprevalence than dogs living in Attica. In the same context, in another study on feline toxoplasmosis in Greece, cats living in Macedonia, an area including Thessaloniki, had a lower seroprevalence than cats living in Central Greece, an area including Attica [2]. These findings suggest lower environmental contamination and *T. gondii* oocyst burden in Thessaloniki and Macedonia than in Attica and Central Greece. In contrast, another recent *T. gondii* seroprevalence study on chickens revealed a higher seropositivity in chickens from Central Greece-Attica compared to those from Central Macedonia, but the difference was not statistically significant [42].

The results of this study also suggest that dogs in both urban and rural regions are exposed to *T. gondii.* Dogs acquire infection by ingesting oocysts, hunting infected prey, or eating undercooked meat [17,18]. The existence of two horizontal transmission routes in dogs may also contribute to the higher seroprevalence in this animal species compared to others that are typically exposed to *T. gondii* through one pathway [17]. For instance, production animals are not omnivores, and cats are not typically coprophagous [43]. It is worth noting that current evidence suggests that most dogs acquire *T. gondii* infection horizontally after birth and not transplacentally [10]. This further supports the theory that coprophagy and rubbing in soil, cat faeces, and foul substances play an essential role in canine infections [6]. Considering that a cat eating an infected mouse can expel more than a billion oocysts during patency [44], it becomes clear that dogs who regularly ingest cat faeces have a considerably increased infection risk [32]. In this frame, based on their high seropositivity, dogs represent great sentinels in Greece for assessing the distribution of *T. gondii* oocysts in the environment [18].

To exacerbate the above, regarding human infection, some studies have identified dog ownership as a potential risk factor, particularly in rural areas and in young children [45,46]. As mentioned earlier, dogs can serve as transport hosts for *T. gondii* oocysts, especially when cats are present in the same household [45]. Acknowledging the high seroprevalence rate of the dogs examined in the current study, canine owners, and particularly young children, should employ hygiene measures to avoid the accidental ingestion of oocysts. These include washing their hands after petting their dogs and after disposing of dog faeces, as well as limiting dogs’ access to cat faeces and litterboxes. Dogs’ fur, limbs, mouth, and faeces may harbour *T. gondii* oocysts [6,10,20,24,25,26], while the oocysts also remain viable after passing through the dog’s gastrointestinal tract. However, it should be noted that the prevalence of *T. gondii* oocysts in dog faeces is expected to be very low [26]. Moreover, dogs should be fed commercial food or properly cooked meat (>60 °C for 10 min) [47], and owners should also control their dogs’ hunting instincts so as not to prey on intermediate hosts (i.e., birds or small mammals) that might harbour *T. gondii* tissue cysts, representing an additional source of infection [9].

## 5. Conclusions

In conclusion, exposure to *T. gondii* is prevalent in the canine population across Greece, with dogs living in rural areas exhibiting a higher infection risk than their counterparts in urban areas. The substantial IgM seropositivity rate of 22.2% suggests that recent or active infections are common in the examined population. Considering the high overall seroprevalence at 47.6%, *T. gondii* appears ubiquitous throughout Greece’s urban and rural regions. Consequently, this high environmental contamination indicates that humans and other animals are at risk of exposure to *T. gondii* oocysts [16]. Therefore, to mitigate the infection risk, dog owners should take preventative measures to avoid accidental ingestion of oocysts, such as washing their hands after petting their dogs and properly disposing of their pet’s faeces. Further hygienic measures, including the thorough cooking of meat intended for dog consumption, are necessitated to reduce the risk of *T. gondii* infection in dogs and safeguard public health.

## Figures and Tables

**Table 1:** *Toxoplasma gondii* IgG, IgM, and overall (IgG or IgM) seroprevalence in dogs from different areas of Greece.

Geographical Location	Number of Tested Dogs	IgG Seroprevalence % (95% CI)	IgM Seroprevalence % (95% CI)	Overall Seroprevalence % (95% CI)
Attica	450	33.8 (29.4–38.1)	23.6 (19.5–27.6)	46.4 (41.9–51.1)
Thessaloniki	354	29.7 (25.1–34.2)	20.1 (15.9–24.2)	40.7 (35.7–45.9)
Other areas of Greece	478	38.3 (33.7–42.4)	22.4 (18.6–26.0)	53.8 (49.3–58.2)
Total:	1282	34.3 (31.8–37.0)	22.2 (20.0–24.5)	47.6 (44.9–50.3)

CI: confidence interval.

## Data Availability

The original contributions presented in the study are included in the article, further inquiries can be directed to the corresponding author.
